# Effects of Intestinal Microbial–Elaborated Butyrate on Oncogenic Signaling Pathways

**DOI:** 10.3390/nu11051026

**Published:** 2019-05-07

**Authors:** Jiezhong Chen, Kong-Nan Zhao, Luis Vitetta

**Affiliations:** 1 Medlab Clinical, Sydney, NSW 2015, Australia; jiezhong_chen@meldab.co; 2 Centre for Clinical Research, The University of Queensland, Royal Brisbane & Women’s Hospital Campus, Herston, Brisbane, QLD 4029, Australia; k.zhao@uq.edu.au; 3 Sydney Medical School, Faculty Medicine and Health, The University of Sydney, Sydney 2006, Australia

**Keywords:** butyrate, short chain fatty acids, intestinal microbiota, cellular signaling

## Abstract

The intestinal microbiota is well known to have multiple benefits on human health, including cancer prevention and treatment. The effects are partially mediated by microbiota-produced short chain fatty acids (SCFAs) such as butyrate, propionate and acetate. The anti-cancer effect of butyrate has been demonstrated in cancer cell cultures and animal models of cancer. Butyrate, as a signaling molecule, has effects on multiple signaling pathways. The most studied effect is its inhibition on histone deacetylase (HDAC), which leads to alterations of several important oncogenic signaling pathways such as JAK2/STAT3, VEGF. Butyrate can interfere with both mitochondrial apoptotic and extrinsic apoptotic pathways. In addition, butyrate also reduces gut inflammation by promoting T-regulatory cell differentiation with decreased activities of the NF-κB and STAT3 pathways. Through PKC and Wnt pathways, butyrate increases cancer cell differentiation. Furthermore, butyrate regulates oncogenic signaling molecules through microRNAs and methylation. Therefore, butyrate has the potential to be incorporated into cancer prevention and treatment regimens. In this review we summarize recent progress in butyrate research and discuss the future development of butyrate as an anti-cancer agent with emphasis on its effects on oncogenic signaling pathways. The low bioavailability of butyrate is a problem, which precludes clinical application. The disadvantage of butyrate for medicinal applications may be overcome by several approaches including nano-delivery, analogue development and combination use with other anti-cancer agents or phytochemicals.

## 1. Introduction

The microbiome has evolved with the human host in a co-dependent manner, with mutual beneficial effects. The microbiome of the intestines comprises the most studied site [1,2]. There are a large number of bacteria in the intestines, reaching concentrations of bacterial cells of the order of 10^11^ cells per millilitre of fluid in the large bowel. These bacteria belong to an estimated 150–400 species with most of them belonging to the *Bacteroidetes*, *Firmicutes*, *Actinobacteria*, *Proteobacteria* and *Verrucomicrobia* phyla. The gut microbiome is subject to high variation depending on dietary practices, life styles, exposure to environment factors and disease states. Manipulation of the gut microbiome has been posited and advanced to improve human health, and for the treatment of diseases [3,4]. For example, fecal microbial transplantation has been used to treat *Clostridium difficile* infections [5]. Gut commensal bacteria can produce vitamins for the host, limit pathogenic bacterial over–growth, stimulate immune responses and secrete SCFAs such as acetate, butyrate and propionate [6]. Importantly, the gut microbiota has been associated with both cancer prevention and treatment.

Many studies have shown that the gut microbiota is closely associated with various cancers that are not only located in the intestines but also in other sites of the body. Dysbiosis, which is caused by the dysregulation of the microbiota, can increase chronic inflammation states and decrease immune responses, leading to an increased cancer incidence [7,8]. In contrast, commensal bacteria can increase immune surveillance and thus decrease cancer incidence. For example, Ma et al. (2018) found that commensal gut bacteria activated natural killer T-cells, which can eliminate cancer cells, through increased CXCL 16 expression stimulated by a change in the primary to secondary bile acid ratio [9]. The intestinal microbiota also plays an important role in cancer therapy efficacy. Recently major discoveries have reported that the gut microbiota is closely associated with cancer immunotherapy, greatly improving patient responses to anti-immune checkpoint agents [10,11,12,13,14,15,16]. Matson et al. (2018) analysed gut microbiota in metastatic melanoma patients and found that *Bifidobacterium longum*, *Collinsella aerofaciens* and *Enetrococcus faecium* were much more abundant in metastatic melanoma patients who responded well to anti-PD-1/anti-PD-L1 immunotherapy [17]. The role of the commensal bacteria in cancer immunotherapy was further confirmed by the experiment in mice which showed that fecal microbiome transplantation (FMT) from responding patients to germ-free mice increased the responses to anti-PD-L1 treatment [17]. Gopalakrishnan et al. (2018) found that *Ruminococcaceae* family was richer in melanoma patients responding to anti-PD-1 immunotherapy [18]. FMT from the responding patients to germ-free mice showed enhanced systemic and anti-tumor immunity. Routy et al. (2018) found that the responding rate to anti-PD-1/anti-PD-L1 was correlated with the abundance of *Akkermansia muciniphila* in cancer patients [19]. FMT from non-responding patients to germ-free mice did not increase anti-PD-1/anti-PD-L1 efficacy in mice but it did if *Akkermansia muciniphila* was administered after FMT, further demonstrating the effect of the bacterial strain. FMT has been used for improving cancer treatment efficacy but some problems remain to be solved.

The beneficial effects of commensal bacteria have been regarded to be mediated at least partially by their metabolites short chain fatty acids (SCFAs) including acetate, butyrate and propionate [20]. Acetate is a molecule with a backbone with two carbons while propionate three carbons and butyrate four carbons. Among the SCFAs secreted by the gut commensal cohort of bacteria in the large intestine, acetate is the most abundant SCFA [20]. However, butyrate is of significant interest given that butyrate provides more than 70% of the energy used for colonocytes and thus is highly important for intestinal physiology. Except providing energy for colonocytes, butyrate has multiple other health promoting effects such as maintaining an intestinal barrier function, reducing inflammation, resisting invasion of pathogens and protection from carcinogenesis [20,21,22].

Furthermore, a diet rich in fiber has been associated with a low risk of developing large bowel cancer because fermentation of the dietary fibers by the intestinal microbiome results in the production of butyrate that has anti-cancer activity [23]. Butyrate has been demonstrated to be the metabolite that exhibits the strongest preventive and therapeutic effects on cancer [24]. Published studies have revealed that butyrate provides a strong anti-cancer role in various cancer cell cultures and mouse models [25]. Many studies have provided evidence that butyrate displays an anti-cancer activity through involvement in different signaling pathways that regulate cell survival and apoptosis in various cancer cells [26]. In this review, we summarize how butyrate exerts anti-cancer effects through modulation of intracellular signaling pathways and discuss the potential implication of butyrate in cancer prevention and treatments as well as associated problems.

## 2. Laboratory Evidence of Anti-Cancer Effect of Butyrate

The anti-cancer effect of butyrate has been well demonstrated in both animal models and cultured cancer cell lines. Donohoe et al. (2014) used a gnotobiotic mouse model to demonstrate the importance of the gut microbiota and butyrate in cancer prevention through the beneficial effects from dietary fiber [25]. In a rat azoxymethane (AOM) model, type III resistant starch (RS), and short chain fructo-oligosaccharides (FOS) which produced a large amount of butyrate decreased aberrant crypt foci while starch free wheat bran (WB), which did not produce butyrate had no observed anti-cancer effects [27]. Clark et al. (2008) also showed in an AOM colon cancer model of rats fed RS and butyrylated-RS that butyrate concentrations were negatively correlated with tumor numbers and size [28]. Tian et al. (2018) showed that administration of SCFA mix, which contains butyrate, acetate and propionate, reduced AOM/DSS—induced colitis-associated colon cancer tumor incidence and size [29]. The tumor cell proliferation was reduced and apoptosis was increased. It also improved colon inflammation and decreased pro-inflammatory cytokines IL-6, TNF-alpha and IL-17 [29]. Bishehsari et al. (2018) found that polyposis in TS4CrexcAPC^lox468^ was associated with decreased SCFA-producing bacteria while supplementation of a high-fiber diet decreased polyp formation through increased SCFAs production [30].

The anti-cancer effect of butyrate has been demonstrated in several cancer cell lines. In a colon cancer cell line HCT116, butyrate promoted apoptosis and increased apoptosis induced by TRAIL [31,32]. Addition of sodium butyrate into cultures of HT-29 and Caco-2 colon cancer cells resulted in apoptosis, decreased cell proliferation, colony formation and cell invasion [33]. The anti-cancer effect of butyrate has also been shown in a breast cancer cell line MCF-7 [34]. Butyrate has been shown to decrease the viability of U937 leukemia cells by 60% [35]. Butyrate can also trigger apoptosis of prostate cancer cell DU145 [26]. Sodium butyrate re-established E-cadherin in ovarian cancer cell line A2780 and increased the sensitivity of these cells to cisplatin treatment [36]. Therefore, butyrate has an effective causal role in the apoptosis of multiple cancer cell types. 

Butyrate has been studied in cancer cell lines in combination with conventional chemotherapeutic agents such as doxorubicin and adriamycin. Butyrate increased the effects of doxorubicin [37]. Butyrate increased adriamycin cytotoxicity through down-regulating human telomerase reverse transcriptase (hTERT) in uterine cancer cells [38]. In Ewing sarcoma cells, butyrate produced a synergistic effect with doxorubicin and etoposide [39]. Butyrate has also been shown to increase effectiveness of irinotecan in colon cancer cells [40]. These results indicate that butyrate could be incorporated into current anti-cancer chemotherapeutic regimens to increase treatment efficacy.

## 3. Butyrate Is a Histone Deacetylase (HDAC) Inhibitor

HDACs are important regulators of genes in post-transcriptional levels and are altered in many cancers [41,42]. Increased expression of HDACs results in decreased acetylation of histones, which is a major component of chromatin, a machine for gene transcription. Histone acylation status affects gene transcription. It is considered that when it is acetylated, regulatory genes are transcriptioned and thus carcinogenesis is inhibited [41]. Inhibition of HDACs can increase histone acetylation and has been extensively studied for cancer treatment [41,42,43,44]. There are 4 classes of HDACs. Class I (HDACs1, 2, 3 and 8), II (IIA 4, 5, 7 and 9; and IIB 6 and 10), IV (HDAC 11) are Zn^2+^-dependent while class III is NAD^+^-dependent (sirtuins 1–7) [41]. 

The mechanisms for the anti-cancer effect of butyrate are considered to be associated with anti-HDAC inhibition. Unlike in colonocytes where butyrate is metabolized in the cytosol, in cancer cells butyrate cannot be metabolized due to the Warburg effect and thus accumulates and enters into the nuclear space where it inhibits histone deacetylation [26]. It has been reported that treatment of cells with sodium butyrate increased histone acetylation by inhibiting deacetylases [45]. Butyrate was also shown to decrease cancer cell proliferation in contrast to increase normal colonocyte proliferation [46]. This epigenetic modification causes hyperacetylation of histone, which regulates numerous enzymes involved in cell survival and apoptosis.

Inhibition of HDAC8 resulted in decreased activity of the JAK2/STAT signaling pathway [47]. Among the pathway, STAT3, a transcription factor encoded by the *stat3* gene, has been extensively studied [48]. Following activation, STAT3 enters the nucleus, leading to increased activities of signaling molecules bcl-2, cyclin D1, c-myc, bcl-xl and Hif, and thus resulting in decreased cell apoptosis and increased proliferation [48,49]. STAT3 also causes activation of angiogenesis factors IL-8 and VEGF [50,51]. Therefore, inhibition of the STAT3 pathway by butyrate in cancer could lead to both increased cell death and decreased angiogenesis. Klampfer et al. (2003) showed that butyrate blocked INF-gamma stimulated the JAK2/STAT1 pathway [52]. Secreted frizzled-related protein (SFRP), a negative modulator of the Wnt signaling pathway, is frequently inactivated in human gastric cancers. Sodium butyrate (NaB) induced demethylation and histone modification at the promoter region of SFRP1/2 restoring the SFRP expression to generate anti-tumor effects in human gastric cancer cells [53]. 

**Figure 1:**
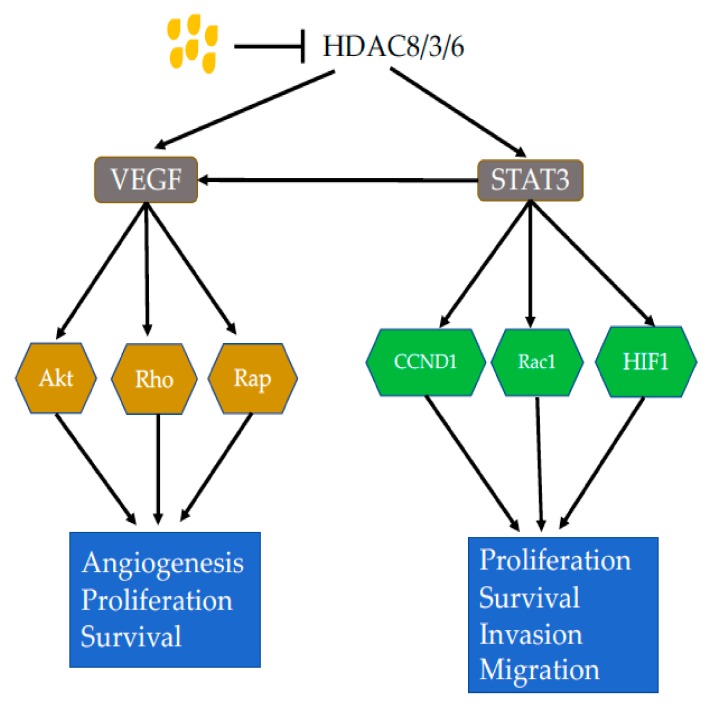
Effects of Butyrate on HDACs. Butyrate inhibits HDAC8, 3 and 6, which leads to decreased expression of VEGF, resulting decreased activities of Akt, Rho and Rap. These signaling molecules regulate angiogenesis, proliferation and survival of cancer cells. Inhibition of HDACs also causes decreased activity of STAT3, leading to decreased expression of Cyclin D1, Rac1 and HIF1, causing decreased cancer cell proliferation, survival, invasion and migration. Abbreviations: Akt, protein kinase B; HDAC, histone deacetylase; HIF1, hypoxia-inducible factor 1; Rac1, Ras-related C3 botulinum toxin substrate 1; Rap, Ras-proximate; Rho, Ras homolog family; STAT3, signal transducer and activator of transcription 3; VEGF, vascular endothelial growth factor.

Vascular endothelial growth factor (VEGF), an angiogenesis factor, plays key roles in carcinogenesis and is increased in many cancers correlated with cancer progression, invasion and metastatic disease [54,55]. Butyrate has been shown to down-regulate VEGF expression [56,57,58]. In colon cancer cell line, butyrate reduced VEGF 165 protein levels in a dose-dependent manner [59]. However, VEGF mRNAs were reduced in a less proportion. In addition, the medium from butyrate-treated Caco2 cells was unable to stimulate HUVEC cell proliferation. VEGF levels were reduced in the medium. The butyrate-caused VEGF reduction has been associated with the down-regulation of upstream HIF [59,60]. VEGF binds to its receptors VEGFR1 and 2 to cause not only an angiogenesis effect but also activation of signaling pathways MAPK and PI3K/Akt [61,62]. Both pathways are important in carcinogenesis with a cascade of down-stream target proteins (Figure 1) [63,64,65,66,67]. PI3K/Akt has a large number of down-stream target proteins that regulate cell proliferation, cell survival, cell migration, genomic instability and immune escape [68,69,70,71]. Butyrate has been shown to reduce neuropilin-1 (NRP-1), which is also a transmembrance receptor of VEGF [56]. NRP-1 is over-expressed in cancer and mediates cell survival and metastasis [72,73,74]. Butyrate decreased NRP-1 at mRNA levels and thus recuced its cancer promoting effect [56]. 

Butyrate has been shown to inhibit the PI3K/Akt pathway through HDAC3 inhibition, leading to inhibition of cancer cell migration [75]. In addition, Bai et al. (2010) proved that butyrate increased PTEN expression, promoted the expression of MUC2 and induced the differentiation of gastric cancer cells [76]. In a mouse model with colitis stimulated by 2,3,6-trinitrobenzene sulfonic acid (TNBS), butyrate reduced inflammation through inactivation of Akt and its downstream NF-κB signaling pathways [77].

In summary, butyrate can inhibit HDACs and thus reduce activities of HDACs-associated oncogenic signaling pathways. This may partially explain anti-cancer effect of butyrate on many types of cancers. 

## 4. Mitochondrial Apoptotic Pathway Altered by Butyrate

**Figure 2:**
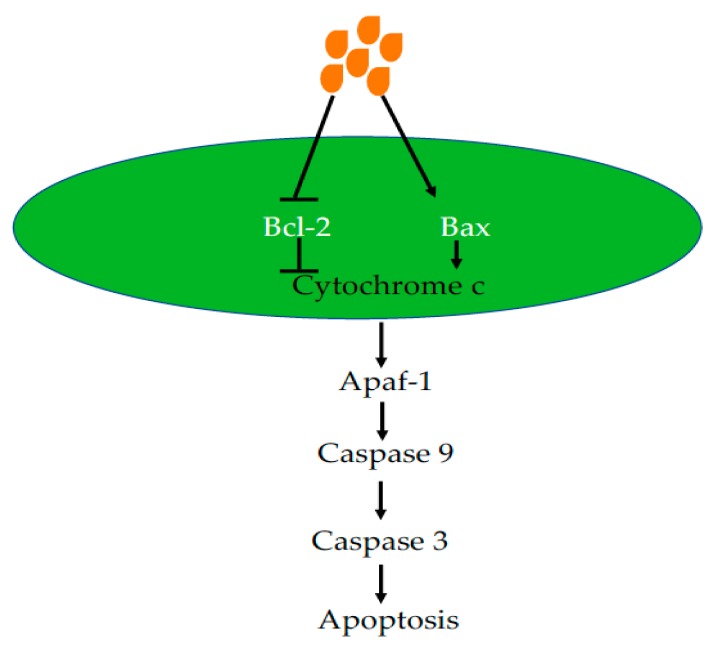
Effects of Butyrate on Mitochondrial Apoptotic Pathway. Butyrate inhibits anti-apoptotic protein Bcl-2 but increases pro-apoptotic protein Bax to cause cytochrome release from mitochondria. This leads to Apaf-1 oligomerization and apoptosome formation, causing procaspase-9 activation and procaspase 3 cleavage for apoptosis. Abbreviations: Apaf-1, apoptotic activating factor-1; Bcl-2, B-cell lymphoma 2; Bax, Bcl-2-associated X protein.

Apoptosis that occurs in multicellular organisms is a process of programmed cell death (Figure 2). Both intrinsic and extrinsic pathways can cause cell apoptosis. The intrinsic pathway is also called the mitochondrial pathway, which is initiated by p53 [78]. Tumor protein p53, a tumor suppressor, controls G1 and G2 checkpoints activating or inhibiting genes involved in the cell cycle, apoptosis and DNA repair [78]. In response to environmental stimuli such as UV and toxins, p53 is increased to promote DNA repair or cause cell death if DNA damage is too severe to repair. In mitochondria, p53 increases the activities of pro-apoptotic proteins such as Bax and decreases the activities of anti-apoptotic proteins such as Bcl-2. The common anti-apoptotic protein include Bcl-2, Bcl-xl and Mcl-1 [79]. Pro-apoptotic proteins include Bak (Bcl-2 antogonist killer 1) and Bax (Bcl-2 associated x protein). An imbalance of the Bax/Bcl-2 ratio causes leakage of cytochrome c, which activates Apaf-1 and caspases 9 and 3, resulting in apoptosis [80]. The mitochondrial apoptotic pathway has been manipulated for cancer treatment such as with the discovery of the Bcl-2 inhibitor ABT-737, which has been extensively tested [80,81,82]. A study has reported that delivery of Bax mRNA by gold nanoparticles could cause apoptosis both in cancer cell lines in vitro and xenograft tumor models in vivo [83].

Butyrate has been demonstrated to cause apoptosis via the mitochondrial pathway in various cell lines. In the fall armyworm *Spodoptera frugiperda* Sf9 cells, butyrate caused mitochondrial apoptotic pathway activation, indicated by an increased Bax/Bcl-2 ratio, cytochrome c release and caspase 3 activation [84]. This has also been demonstrated in cancer cells, treatment of prostate cancer cell DU145 and PC3 caused decreased growth and increased apoptosis with decreased anti-apoptotic protein Bcl-xl and Bcl-2 and increased pro-apoptotic protein Bax and Bak [85]. Previous studies have also shown that butyrate can induce apoptosis in colon cancer cells (Caco-2), but not in normal intestinal epithelial cells, with this effect being mediated via the mitochondrial pathway and inducing a clear shift of the mitochondrial Bcl-2 rheostat towards an array of pro-apoptotic effects [86,87]. The increase in apoptosis was associated with the up-regulation of the caspase cascade including DNA fragmentation, pro-apoptotic Bax expression and translocation of cytochrome-c from the mitochondria to the cytosol and down-regulation of anti-apoptotic Bcl-2 and Bcl-xl in different human cancer cells [86,87,88,89]. The caspase cascade was activated via the formation of an apoptosome that includes caspase-9 and two other key executioner enzymes caspases-3 and -1 [86,87]. In addition, butyrate targets peroxisome proliferator-activated receptor gamma (PPARγ) a type II nuclear receptor that activates caspase-3, increases caspase-8 and -9 activity and reduces expression of XIAP and survivin leading to apoptosis in colorectal cancer cells (Caco-2 cells) [90]. Addition of sodium butyrate into osteosarcoma cells increased cell apoptosis and decreased cell proliferation, resulting in increased Bax and decreased Bcl-2 through an increase of p53 and a decrease of MDM2 [91]. Sodium butyrate also caused a decrease in the level of dynamin-related protein 1 (DRP1) in HCT116 and SW480 colon cancer cells, indicating decreased mitochondrial fission [92]. In addition, both survivin and Bcl-2 expression was decreased. Butyrate reduced mitochondrial protein Bcl-2 levels, together with a decrease of chaperone protein GRP78, which is involved in endoplasmic reticulum (ER) stress signaling in HL-60 cells [93]. 

## 5. Butyrate-Induced Extrinsic Cell Death Pathway

**Figure 3:**
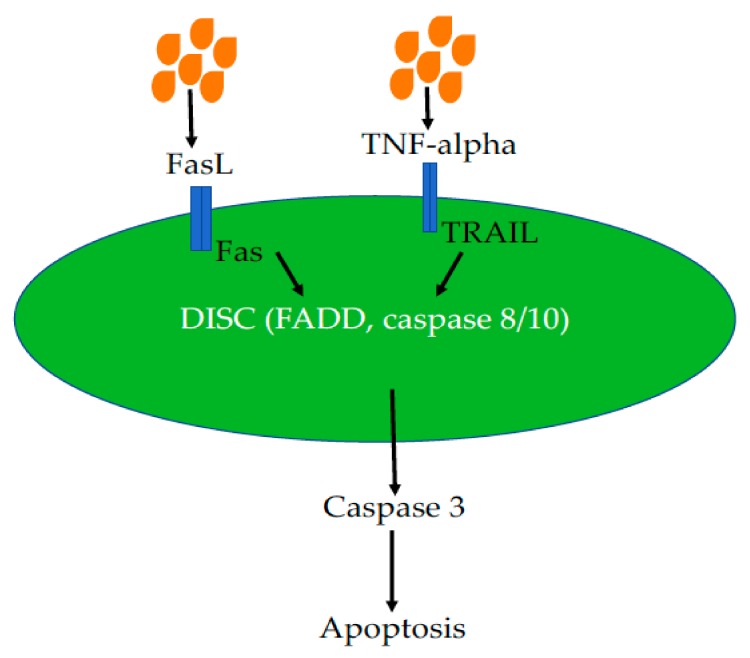
Effects of Butyrate on Cell Death Pathway. Butyrate acts both FasL/Fas and TNF-alpha/TRAIL systems of the cell death pathway to cause the formation of DISC, which includes death receptor, FADD and caspase 8, leading to caspase 3 activation and apoptosis. Abbreviations: DISC: death-inducing signaling complex; FADD, Fas-associated protein with death domain; Fas L, ligand of death receptor Fas; TRAIL, tumor necrosis factor-related apoptosis-inducing ligand.

In breast cancer MCF-7 cells, butyrate inhibited cell growth to induce apoptosis via the transmembrane protein Fas (first apoptosis signal, CD95) and potentiated Fas-triggered apoptosis in a p53-independent manner [94]. Apparently, butyrate also induces extrinsic cell death pathways. In one extrinsic pathway, the death-inducing signaling complex (DISC) is formed, which contains FADD, caspase 8 and caspase 10 when Fas bound by Fas ligand [95,96] (Figure 3).

Butyrate increased Fas promoter activity by inhibiting HDAC bound, leading to hyperacetylation of the promoter and increased transcription. Thus, butyrate could cause T cell apoptosis through upregulation of Fas [97]. In addition, TRAIL and TNF bind to death receptors (DR4 and DR5), resulting in activation of caspase 8. Activated caspase 8 in turn cleaves caspase 3 to cause apoptosis [98]. Kim et al. (2004) found that butyrate increased DR5 expression in HCT-116 cells and increased the sensitivity of HCT116 cells to TRAIL-induced apoptosis [31]. This finding provides new insights into how butyrate suppresses colon carcinogenesis in HCT116 cells. A later study has further proved that butyrate increases DR4/5 expression in colon cancer cells [99]. Furthermore, the store-operated Ca^2+^ entry (SOCE) pathway plays a key role in both normal cells and cancerous cells. Butyrate triggers colon cancer cell apoptosis in a SOCE-dependent manner [100]. These findings may open interesting perspectives for the use of butyrate in novel human cancer treatment strategies.

## 6. Butyrate Receptors GPR41, GPR43 and GRP109a Signaling Pathways

Butyrate can exert its effects through its receptors-mediated signaling pathways. There are three receptors which bind SCFAs, namely GPR41, GPR43 and GPR109a [101,102,103,104,105]. These receptors are located on the cell surface. When they are activated, oncogenic signaling pathways are inhibited. It has been demonstrated that Knockout of GPR109a in mice (*Gpr109a*^−/−^) increased colon cancer incidence indicated by increased polyps in an AOM/DSS model as well as in APC^min/+^ mice [101,103]. Antibiotics treatment of mice, which greatly reduced fermentation, increased polyp number and size in an AOM/DSS model of colon cancer, was prevented by supplementation of GPR109a activator niacin [101]. In addition, overexpression of GPR109a in a cultured cancer cell line with addition of its activator caused apoptosis [101]. GPR43 knockout has also been shown to increase colon cancer development in an Apc^min/+^/DSS model [106]. 

**Figure 4:**
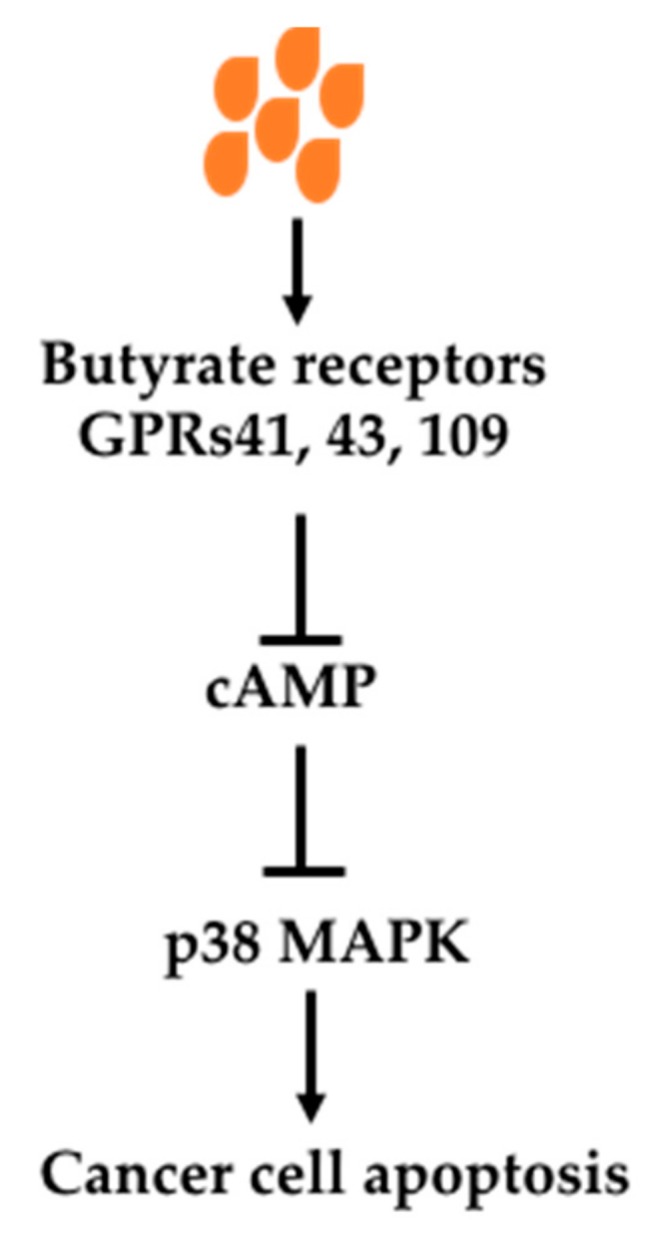
Effects of butyrate on signaling molecules through its receptors. Butyrate caused decreased cAMP through its receptors GP41, GP43 and GP109, resulting in activation of p38 MAPK pathway and cancer cell apoptosis. Abbreviations: cAMP, cyclic adenosine 3′,5′-monophosphate; GPR41, G-protein coupled receptor 41; GPR43, G-protein coupled receptor 43; GPR109, G-protein coupled receptor 109; p38 MAPK, p38 mitogen-activated protein kinases.

The signaling pathway has been shown to be mediated by cAMP and p38 MAPK (Figure 4). In a breast cancer cell line, binding of butyrate to its receptors upregulates intracellular Ca^2+^, reduces forskolin-induced cAMP levels, increases phosphorylation of mitogen-activated protein kinase (MAPK) p38 and heat shock protein 27 (HSP27) activation, causing apoptosis. The p38 MAPK pathway induced by butyrate was verified in prostate cancer cell line Du145 with increased upstream enzymes MAPK kinase 3 and 4 (MKK3 and MKK4) [88]. In a colon cancer cell (HCT116), butyrate caused cell cycle arrest and apoptosis through increased p21 and decreased pERK1/2 while it increased pERK1/2 in a noncancerous colon cell line (NCM460) [107]. It has been shown in HT-29 and HCT8 cells, GPR3 activation caused cell cycle arrest and increased p21 levels and decreased levels of cyclin D3 and cyclin-dependent kinases (CDKs) 1 and 2 [108]. Thus, butyrate-caused cancer cell cycle arrest and apoptosis through binding to its receptors GPR41, GPR43 and GPR109a on cancer cell surface and associated multiple signaling changes.

## 7. Effect of Butyrate on Wnt Signaling Pathway

**Figure 5:**
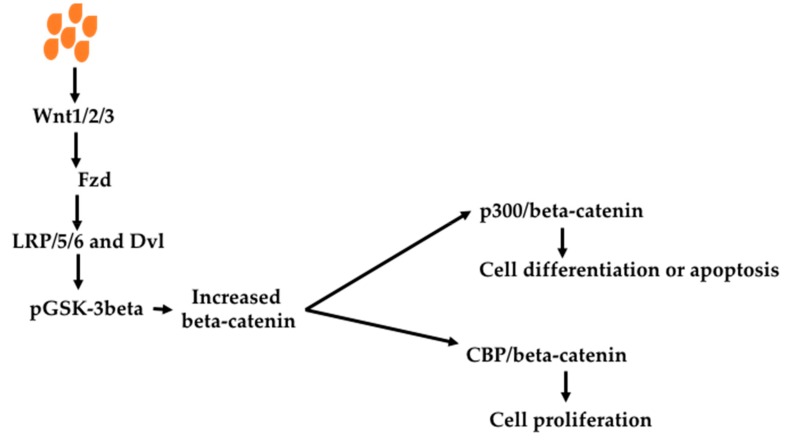
Effect of butyrate on the Wnt signaling pathway Butyrate can increase Wnt1/2/3 expression, which bind to their receptor Fzd to activate Wnt signaling pathway. Thus, Fzd activates LRP5/LRP6/Dvl complex, which phosphates GSK-3beta. This reduces beta-catenin degradation, causing accumulation of beta-catenin. When beta-catenin binds to p300 protein, it causes cell proliferation or apoptosis. When beta-catenin binds to CBP, it causes cell proliferation. Abbreviations: CBP, cAMP response-element binding protein binding protein; Dvl, disheveled protein; Fzd, Frizzled; GSK-3beta, glycogen synthase kinase–3 beta; LPR5, low-density lipoprotein receptor- related protein 5; LPR6, low-density lipoprotein receptor-related protein 6; Wnt1/2/3, wingless-related integration.

The Wnt signaling pathway is associated with many cancers [109,110,111,112]. It is initiated by protein Wnt binding to its receptor Fzd, leading to formation of Fzd/LRPS5/6/Dvl. Subsequently GSK-3beta is phosphorylated, leading to decreased beta-catenin degradation by GSK-3beta. Accumulated beta-catenin interacts with target genes. Two consequences have been identified—cell differentiation/apoptosis when binding with p300 and cell proliferation when binding with CBP (Figure 5).

Several studies have shown that butyrate can hyperactivate the Wnt signaling pathway and cause colon cancer cells death [113,114]. Histone acetylase p300 can affect Wnt pathway to promote cell differentiation. Therefore, it is not surprising that knockout of p300 causes butyrate-resistance while re-introduction of p300 re-sentilizes colon cancer cells to butyrate [115]. Small molecule inhibitor ICG-001 disrupts association of CBP with beta-catenin, decreasing the cell proliferation effect of the Wnt pathway [116]. The available beta-catenin can bind to p300 and thus inceases cell differentiation effect of the Wnt pathway [117,118]. Manegold et al. showed that ICG-001 increased the sensitivity of pancreatic cancer cells to chemotherapeutic agent gemcitabine with increased let-7miRNA, decreased k-ras and survivin [119]. ICG-001 and BCR-ABL tyrosine kinase inhibitor imatinib combination use in NOD/SCID2Rγ(−/−) mouse model of engrafted human chronic myelogenous leukemia eliminated engrafted leukemia-initiating cells [118].

## 8. Butyrate-Promoted Cell Differentiation through Protein Kinase C Pathway

Protein kinase C (PKC) is a serine/threonine protein kinase that expresses multiple isoforms and is known to be involved in different cellular signal transduction pathways, which mediate cellular functions such as proliferation and differentiation [120,121,122,123]. Lower differentiation is one of hallmarks of cancer. PKC is now generally regarded as a tumor suppressor to promote cancer cell differentiation [124,125,126]. PKCs have been classified into three groups: calcium-dependent cPKCs(α, βI, βII, and γ), “novel” PKCs-nPKCs (δ, ε, η, and θ) and “atypical” PKCs-aPKCs (ζ and ι) [127,128,129]. Choi et al. over-expressed PKCβ1 in HT29 colon cancer cells, which resulted in decreased cell growth and loss of anchorage-independent growth in soft agar [130]. In this study, it has also been shown that butyrate induced differentiation of HT29 cells but not HT29 with overexpression of PKC. 

Sodium butyrate significantly stimulated PKC activation to induce differentiation and turnover in different human cells especially in colon cancer cells [131,132,133,134,135]. Promotion of cancer cell differentiation is one of butyrate’s anti-cancer properties. Notably it upregulates PKCƹ, but down regulates PKCβ during erythroid differentiation. Thus, it is obvious that certain PKC isoforms may play important roles in the signal transduction mechanisms of butyrate, leading to regulation of erythroid proliferation and differentiation [131]. In colon cancer cells with pre-treatment with butyrate, cholinergic stimulation or phorbolester treatment enhances activation of calcium-dependent PKC to induce an increase in membrane-bound cPKC activity and radically reduce expression of distinct high- molecular CD44 variant transcripts v3 (670 bp), v5 (940 bp) and v8 (535 bp) [134]. In addition, McMillan et al. (2003) found that butyrate and the secondary bile acid ursodeoxycholic acid (UDCA) induced apoptosis of human colon adenoma cells through differential activation of PKC and MAPK pathways [136]. Butyrate activated PKCδ and p38 MAPK while UDCA stimulated activation of PKCα and p42/44 MAPK. Butyrate treatment also resulted in the caspase-3-mediated proteolysis of PKCδ. Butyrate-induced apoptosis was reduced by inhibitors of PKCδ, p38 MAPK and caspase 3 such as Rottlerin, SB202190 and DEVD-fmk, in contrast to that UDCA induced proliferative/survival effects were blocked by inhibitors of PKC-α and MEK1 including Gö6976 and PD98059 [136]. The PKC pathway plays an important role in butyrate-mediated cell differentiation and thus cancer prevention. The role of different forms of PKC in the process warrants further studies.

Orchel et al. (2005) tested the effects of butyrate on colon cancer cell lines Caco-2 and HT-29 [133]. At concentration of 1 mM, NaB induced cancer cell differentiation, which was PKC and JNK dependent. Alkaline phosphatase (ALP) was used as a differentiation marker. Inhibition of MEK-ERK increased the effect of butyrate on cancer cell differentiation. At higher concentration (5mM, 10 mM), butyrate caused apoptosis of colon cancer cells. Treatment of Ewing sarcoma cell lines with butyrate also increased differentiation neuronal marker βIII-tubulin [137]. Therefore, PKC-mediated cancer cell differentiation could be another mechanism for the anti-cancer effect of butyrate.

## 9. Butyrate-Mediated Anti-Inflammatory Effect

Butyrate has displayed a host of chemo-preventative properties including not only increased apoptosis, inhibition of cell proliferation, down regulation of angiogenesis, but also enhanced immune-surveillance and anti-inflammatory effects in colorectal cancer cells in vitro [138]. In the intestines, butyrate has been shown to decrease inflammation. The initial finding is that *Clostridia* can induce T-reg cell differentiation. It can increase T-reg cell generation and differentiation in vitro and in vivo [139,140] and thus inhibit activities of proinflammatory immune cells such as CD4 T-cells and CD8 T-cells. Butyrate-induced T-reg ameliorated inflammation, which caused the transfer of CD4^(+)^CD45RB^(hi)^ T cells in Rag^(−/−)^ mice. Thus, butyrate is believed to modulate host immune responses [141]. Butyrate directly modulates human dendritic cell (DC) function by reducing the frequency of peptide-specific CD8^+^ T cells and inhibiting production of IL-12 and IL-23 [141]. In a randomized, double-blind placebo-controlled clinical trial, Luceri and colleagues (2016) have proved that the administration of sodium butyrate enemas could prevent mucosal inflammation and atrophy and affect gene expression profiles thus improving the recovery of tissue integrity in patients after ileo/colostomy [142]. Miyachi et al. (1999) found that butyrate augmented interferon-alpha-induced S phase accumulation and persistent tyrosine phosphorylation of cdc2 in K562 cells. They proposed that a clinical application butyrate combined with IFNα should show significant improvement of clinical effects of IFNα against CML cells [143]. 

It has also been reported that n-butyrate vastly induces many components of eicosanoid signaling pathway including prostaglandin E2, 15d-prostaglandin J2, cyclooxygenase-2, leukotriene B4 and thromboxane B2 in monocytes following TLR4 and TLR2 activation, revealing the role of n-butyrate as a crucial mediator of gut-specific immunity [144]. In addition, butyrate has been shown to decrease ROS in the intestine and thus decrease NF-κB [145]. NF-κB a well-known pro-inflammatory signaling molecule, plays a major role in the control of immune responses and inflammation [146]. NF-κB is increased in about 40% of colon cancers [147]. Butyrate not only suppressed NF-κB activation, but also modulated the activity and expression of the Peroxisome-Proliferator-Activated-Receptor gamma (PPARγ) and the vitamin D receptor (VDR) in colorectal cancer cells [146]. The study suggested that both the nuclear hormone receptors (PPARγ) and vitamin D receptor (VDR) were involved in butyrate-mediated inhibition of inducible NF-κB activation [146]. Butyrate activated T-reg cells also inhibit IL-6 and IL-6 production and thus reduce their down-stream signaling pathways [148].

## 10. Effects of Butyrate on MicroRNAs

MicoRNAs (miRs) are non-coding RNAs which are about 19–25 nucleotides and regulate post-transcripts of DNAs [149,150,151,152]. So far, there are more than 1000 miRs identified in humans, which regulate more than one third gene expression. Therefore, miRs are involved in many physiological processes and dysregulation of miRs is associated with many diseases. Abnormalities in miRs are closely associated with many cancers [153,154,155,156,157]. MiRs that promote activation of oncogenic signaling pathways are called oncomirs while that reduce oncogene expression are called anti-oncomirs [149].

**Figure 6:**
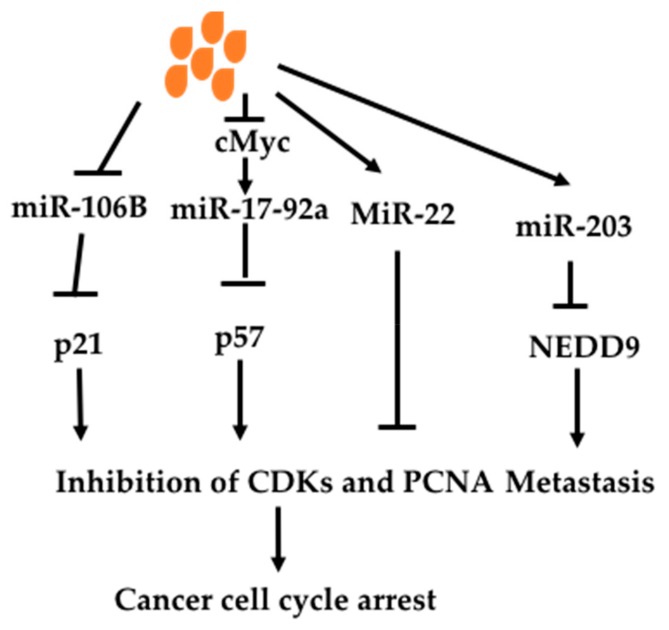
Effects of butyrate on miRNAs. Butyrate can inhit miR-106B, leading to increased expression of p21 protein. It is known that p21 can cause cancer cell cycle arrest through inhibition of CDKs and stop DNA replication through inhibition of PCNA. Butyrate also decreases miR-17-92a through inhibition of cMyc, leading to increased expression of p57. P57 has similar effects to those of p21. Butyrate increases tumor suppressors miR-22 and miR-203. MiR-22 decreases cell cycle while miR-203 reduces metastasis through acts on NEDD9. Abbreviations: CDKs, cyclin-dependent kinases; NEDD9, Neural precursor cell expressed developmentally down-regulated protein 9; PCNA, proliferation cell nuclear antigen.

Butyrate has been shown to regulate miRs to affect oncogenic signaling pathways (Figure 6). Hu et al. (2011) found that butyrate changed 44 miRs in colon cancer cell line HCT-116 [158]. Among them, butyrate decreased an oncomir miR106B. MiR106B is increased in colon cancer, which targets tumor suppressor p21, leading to decreased p21. P21, cyclin-dependent kinase inhibitor 1 or CDK-interacting protein 1; and inhibits all cyclin/CDK complexes such CDK2 [159]. P21 can also act on proliferating cell nuclear antigen (PCNA) to inhibit DNA replication directly [160,161]. P21 mediates cell-cycle arrest caused by p53 activation under DNA damage. P53 binds to p21 promoter to increase p21 expression, causing cell cycle arrest. p21 is also regulated by other transcriptional factors and miRs independent of p53 [162,163]. Thus butyrate can exert an anti-cancer effect through increasing p21 via decreasing miR106B. Xiao et al. (2018) studied miRs altered by butyrate in non-small cell lung cancer cell line A549 and found 33 miRs upregulated and 22 miRs down-regulated [164]. Among them, miR-3935 and miR-574-3p were most upregulated. Overexpression of miR-3935 in A549 resulted in decreased cell proliferation and migration. It was also found to down-regulate oncoprotein RNF115 expression.

c-Myc is an oncogene, which is important in carcinogenesis. c-Myc can induce miR-17-92a to mediate its oncogenic effect. Butyrate has been shown to decrease c-myc-induced miR-17-92a [165]. MiR-17-92a was shown to be seven-fold higher in sporadic colon cancer tissues compared with adjacent normal tissues. Butyrate decreased pri-miR-17-92a, precursor and matured miR-17-92a in c-Myc dependent fashion. Mutation of c-myc promoter or over-expression of c-Myc protein diminished the effect of butyrate on the oncogenic effect of miR-17-92a. Over-expression of miR-17-92a inhibited butyrate-induced p57(Kip2) expression. P57(Kip2) is a known cyclin-dependent kinase inhibitor, which causes cell cycle arrest [166]. P57 is not mutated in cancer but decreased expression is found through epigenetic regulation and is thus used as a prognostic marker and considered as a potential therapeutic target [167].

Butyrate has been shown to increase an anti-oncomir miR-203 and miR-22 [33,168]. Han et al. (2016) found that miR-203 was induced by butyrate in several colon cancer cell lines, leading to decreased cancer cell migration and invasion [33]. MiR-203 is a tumor suppressor, which decreases many oncogenic molecules. In Han’s study, miR-203 was shown to down-regulate NEDD9, a scaffolding protein belonging to the Cas family. It is a biomarker of invasion, migration and prognosis of many types of cancers including lung, breast, liver, gastric colon cancer and melanomas [169]. Pant et al. revealed that miR-22 was increased in hepatic cancer cell line Huh 7 by butyrate [168]. Over-expression of miR-22 resulted in inhibition of HDAC SIRT-1 pathway and ROS production, leading to apoptosis.

## 11. Butyrate and Methylation

Gene promoter methylation is one regulatory mechanism for gene expression [170,171]. Methylation usually happens on cytosine-phosphate-Guanine (CpG) islands to shutdown the promoter. Hypomethylation of oncogenes is common, which leads over-expression of these genes and associated oncogenic signaling pathways [172]. Hypermethylation of tumor suppressor genes can also cause cancer due to decreased negative regulation of oncogenic signaling pathways [173,174]. Modulation of methylation status of oncogenes or tumor suppressors is of therapeutic importance [172,175].

**Figure 7:**
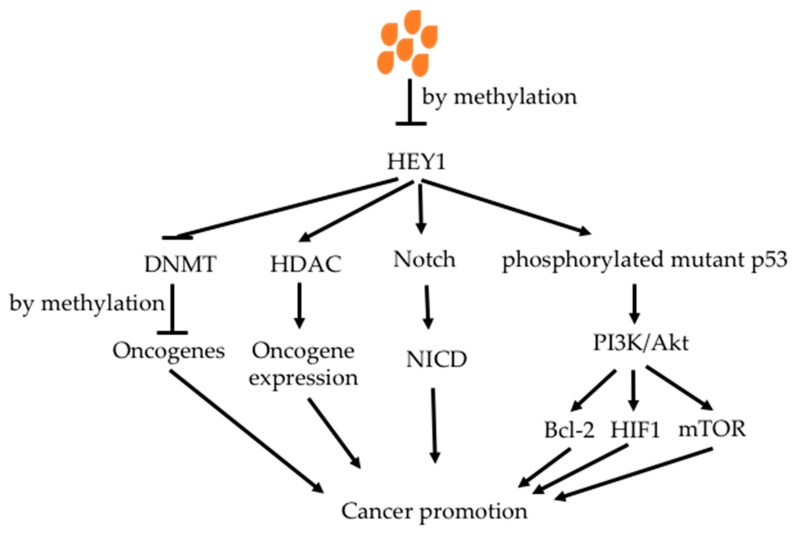
Effect of butyrate on methylation of oncogene HEY1. Butyrate reduces HEY1 expression through increasing the methylation of its promoter. This leads to decreased phosphorylation of mutant p53; mutant p53 can increase PI3K/Akt pathway to promote cancer initiation and progression. It also decreases Notch and HDAC activities. DNMT is increased, which increases methylation of oncogenes, reducing their expression. Abbreviations: Bcl-2, B-cell lymphoma 2; DNMT, DNA methyltransferase; HEY1, Hes related family BHLH transcription factor with YRPW motif 1; HIF1, hypoxia-inducible factor 1; mTOR, mammalian target of rapamycin; NICD, intracellular domain of the notch protein; PI3K/Akt, phosphoinositide 3-kinase/Protein kinase B.

Butyrate has been demonstrated to increase methylation of oncogenes, reducing the activity of oncogenic signaling pathways [176,177,178]. Tsung et al. showed that butyrate increased methylation of oncogene HEY1 in glioblastoma cell lines, decreasing HEY1 expression with increased cell apoptosis and decreased cell proliferation (Figure 7) [179]. HEY1 down-stream proteins were decreased. It also inhibited phosphorlated mutant p53 protein, which is a key factor in glioblastoma.

## 12. A Summary of Butyrate on Multiple Signaling Pathways—Blocking Feed-forward Regulation

Signaling pathways in cancer are very complicated. They are not acting independently but crosstalks are extensive so that activation of multiple signaling pathways can accelerate each other. This leads to higher activation of signaling pathways when they are examined individually. 

**Figure 8:**
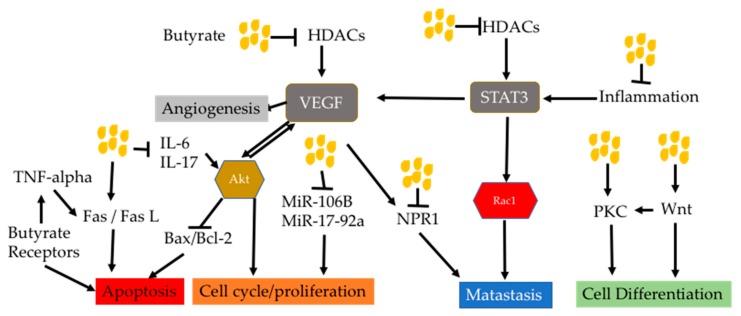
Crosstalk of signaling pathways under butyrate influence. Butyrate can inhibit VEGF through HDACs, which results in decreased VEGFR-mediated angiogenesis. Decreased VEGF also leads to decreased cell-cycle/cell proliferation mediated by Akt and metastasis by its receptor NPR-1. Decreased Akt also results in increased Bax/Bcl-2 ratio and thus increased apoptosis. Decreased Akt could also contribute decreased IL-6 and IL-17 by butyrate. Apoptosis could also be caused by signaling from Fas/FasL and butyrate receptors GPRs. GPRs crosstalks with Fas/FasL through promoting TNF-alpha secretion. They also activate p38 MAPK to cause apoptosis. Butyrate can also block MiR-106B and MiR-17-92a to decrease cell cycle/cell proliferation. Inhibition of HDACs and inflammation by butyrate result in decreased activity of the STAT3 pathway, which also contributes to cell proliferation, survival and metastasis through its downstream. Butyrate activates PKC and Wnt to cause cell differentiation. Wnt can also activate the PKC pathway to form crosstalk. Abbreviations: Akt, protein kinase B; Bcl-2, B-cell lymphoma 2; Bax, Bcl-2-associated X protein; HDACs, histone deacetylase; IL-6, interleukin 6; IL-17, interleukin 17; NPR1, neuropilin 1; PKC, protein kinase C; Rac1, Ras-related C3 botulinum toxin substrate 1; STAT3, signal transducer and activator of transcription 3; TNF-alpha, tumor necrosis factor-alpha; VEGF, vascular endothelial growth factor.

Butyrate can act in multiple signaling pathways which facilitate blockage of such a feed-forward effect. This coud be an advantage of butyrate in anti-cancer use. A figure below showed how signaling pathways targeted by butyrate interact each other (Figure 8). For examples, Akt is activated by VEGF, which is activated by inhibition of HDACs by butyrate. Akt in turn also activates VEGF. Akt has been demonstrated to inhibit mitochondrial apoptotic pathway through regulating Bax/Bcl2 expression. This means that butyrate may regulate the mitochondrial pathway through HDACi. 

Activation of GRP 41 in HepG2 cells by SCFAs has been shown to increase apoptosis through increased secretion of TNF-alpha [180]. This indicates that there is a crosstalk between butyrate receptors and extrinsic death pathway. Meanwhile, the non-canonical Wnt pathway activated by butyrate caused cell differentiation through calcium/PKC pathway [181,182,183].

## 13. Implications 

**Figure 9:**
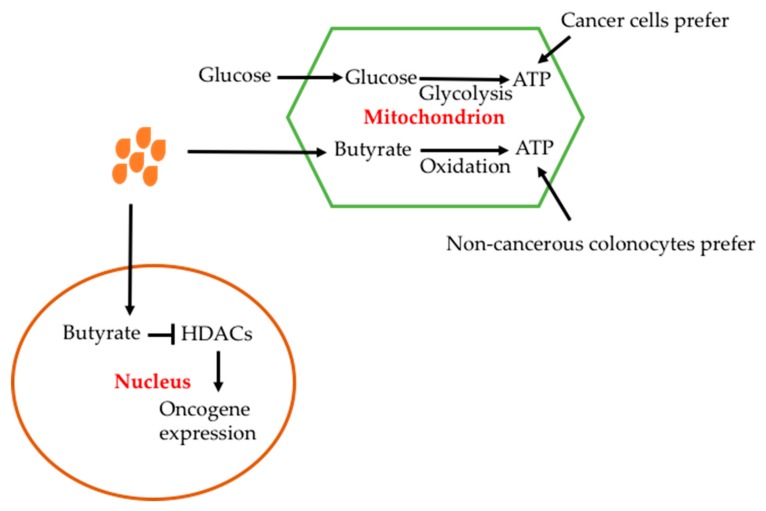
Effects of butyrate on mitochondrion and nucleus. Butyrate can enter mitochondrion, which undergoes oxidation to produce ATP while glucose produces ATP through glycolysis. The pathways are competitive. In cancerous colonocytes, glycolysis is preferred while butyrate oxidation is preferred in normal colonocytes. Butyrate can enter to nucleus to inhibit HDACs, thus oncogene expression is reduced.

The effect of butyrate is cell-type dependent. The advantage for butyrate to be used in cancer therapy is that it has a toxic effect on cancer cells but is beneficial for non-cancerous cells. The different effects have been explained by the Warburg effect of cancer cells; i.e. glucose utilization is increased in cancerous cells (Figure 9) [46,184,185,186]. In colonocytes, butyrate is oxidased to produce ATP; in cancer cells butyrate cannot be metabolized due to the Warburg effect and thus accumulates and enters into the nucleous where it inhibits HDACs [26]. In colorectal cancer cells, butyrate has been shown to decrease the expression of short chain acyl-CoA dehydrogenase, which catalyses oxidation of butyrate [187,188]. 

The effect of butyrate is also dose-dependent. In low concentrations (0.5–1 mM), butyrate promotes non-cancerous colonocytes proliferation but cancerous colonocytes apoptosis [184]. In high concentrations (greater than 2 mM), butyrate can cause both non-cancerous colonocytes and cancerous colonocytes apoptosis [189]. 

Butyrate-mediated signaling inhibition not only results in decreased cancer cell proliferation and increased cancer cell apoptosis, but also increases immune responses to cancer cells. Butyrate can increase immune responses through the interactions with neutrophils and modulate recruitment, effector functionalities and survival in different tissues. It may also mediate microbiota’s role in anti-PD-L1/anti-PD1 cancer immunotherapy. It has been demonstrated that the gut microbiota plays an important role in the treatment response to anti-PD-L1/anti-PD1 therapy. Mice that express a less abundant and variable gut bacteria profile respond poorly to immunotherapies; with responses enhanced following transplantation of a microbiome from patients who responded well but not that from poorly responding patients [19]. Signaling pathways such as PI3K/Akt, Stat3, NF-κB and HIF-1 play key roles in expression of PD-L1 [70], butyrate has been shown to inhibit these signaling pathways, which may result in decreased PD-L1 expression and increased anti-cancer immune responses.

Although butyrate has been shown to have beneficial effects on decreasing inflammation and carcinogenesis, its clinical implications are prevented by its short half-life in the circulation and thus its bioavailability is very low. The use of solid lipid nanoparticles has increased the anti-cancer efficacy of butyrate on HL-60 but not on MCF-7 cancer cells [190]. Nano-delivery has been used to increase the concentrations of anti-cancer drugs in the cancer site to improve treatment efficacy. Compared to conventional drug delivery approaches, nanoparticle–mediated delivery of anti-cancer drugs brings several remarkable advantages. First, drugs delivered by nanoparticles may have a longer biological half-life due to the protection afforded from blood enzymes and as such the possibility of releasing concentrated levels at the site of the cancer due to enhanced permeability and retention (EPR) at the cancer sites [191]. EPR is caused by the leakiness of tumor vasculature as well as poor lymphatic drainage [191]. Therefore, it increases treatment efficacy and decreases side-effects. For example, Doxil (Janssen Biotech), which is doxorubicin formulated in liposomes containing polyethylene-glycol has a 100 times longer circulation half-life and seven-fold lower cardio-cytotoxicity than free doxorubicin [192]. Second, a nanoparticle can contain multiple drugs and thus will facilitate combinatorial therapy. It is known that combination of chemotherapy with immuno-therapy or targeted therapy produces much more effective treatment outcomes. Third, nanoparticles can protect drugs made from fragile siRNA or proteins from biochemical degradation in the human body. This is due to the stealth-like features of nanoparticles [193]. 

Minelli et al. (2012, 2013) have developed Cholesteryl butyrate solid lipid nanoparticles (cholbut SLN) as a delivery system for the anti-cancer drug butyrate [194,195]. In vitro cancer cell experiments, with the cholbut SLN inhibited migration of cancer cells and substantially down-modulated ERK and p38 phosphorylation. There was also tumor cell viability inhibition, clonogenic activity, Akt phosphorylation and cell cycle progression, induced E-cadherin and inhibited claudin-1 expression. In mice model in vivo experiments, the cholbut SLN prevented the adhesion of polymorphonuclear cells to the endothelium, substantially delayed tumor growth and prevented metastases to the lung [194,195].

Butyrate has been used together with epigallocatechin gallate (EGCG) to increase treatment efficacy in cancer cells [196]. This has reduced the dosages used by both agents. EGCG, which has been studied extensively in cancer prevention, is a phytochemical extracted from green tea [197]. Phytochemicals are well known for their effects on cancer prevention, reducing cancer risk by as much as 20% [198]. EGCG in cancer prevention has been studied in both *Apc*^min/−^ and AOM colon cancer animal models. EGCG reduced intestinal tumor formation in *Apc*^min/−^ mouse model [199,200] and reduced polym formation in a AOM model of colon cancer [201,202]. It is plausible then that EGCG could be incorporated with probiotic formulations to increase butyrate effectiveness. Butyrate has been tested for combination application with ABT737 in HL-60 cells, resulting in synergistic effect when butyrate was used at high concentrations (2–5 mM). ABT737 alone caused apoptosis rapidly while butyrate caused cell cycle arrest at G2 first, then caused apoptosis at a later stage. Butyrate has been used together with artemisinin in studies with leukaemia cancer cells [203].

Another approach to increase the effective application of butyrate is to select more effective analogues. Structure-activity relationships of butyrate analogues have been studied [204]. Among numerous butyrate derivatives, 4-phenylbutyrate and tributyrin are the most effective [205,206]. 

Modulation of the intestinal microbiota could be an attractive approach to increase butyrate production in the intestines. The advantage of this approach is that the intestinal microbiota can continuously produce levels of butyrate. Specific intestinal bacteria have been reported to produce butyrate; the two most important bacterial species inlcude *Faecalibacterium prausnitzii* (from the Clostridial cluster IV) and *Eubacterium rectale* (from the Clostridial cluster XIVa), which account for 14% and 13% of the total faecal gut microbiota respectively [207]. Other butyrate-producing bacterial species include *Roseburia* spp. (Clostridial cluster XIVa, namely *Roseburia faecis, Roseburia inulinivorans, Roseburia intestinalis*, and *Roseburia hominis*), *Eubacterium* spp. (Clostridial cluster XIVa, namely *Eubacterium hallii*), *Anaerostipes* spp. (Clostridial cluster XIVa, namely *Anaerostipes butyraticus, Anaerostipes caccae*, and *Anaerostipes hadrus*), and *Butyricicoccus pullicaecorum* (*Clostridial cluster IV*) [208]. It has not been known whether several species such as *E. rectale*, *F. prausnitzii*, and *R. intestinalis* [209] that preferentially colonize the mucus layer, and thus increase the butyrate bioavailability for colon epithelial cells, have better anti-cancer effects than *A. caccae* that is mainly found in the lumen of the colon.

Several phytochemicals have been shown to increase the production of butyrate by the gut microbiota. Different phytochemicals that are used by various bacteria are selective [97]. Phytochemicals that promote butyrate-producing bacteria include resistant starch and oligosaccharides [210]. Therefore, these phytochemicals have been used as food supplementation to increase butyrate production.

## 14. Conclusions

Butyrate has been shown to have anti-cancer effects both in in vitro cancer cell culture systems and in in vivo animal model experiments. The associated mechanisms involve many signaling pathways as well as anti-inflammatory actions through T-reg cell maturation. Butyrate can inhibit HDACs and thus decrease the activities of their downstream signaling pathways VEGF and STAT3. Butyrate also acts on both mitochondrial and extrinsic cell death pathways to cause cancer cell apoptosis. Through anti-inflammation, butyrate inhibits IL-6 and IL-17 affects STAT3 and NF-κB signaling pathways. Butyrate can also regulate miRs and methylation to alter signaling molecules. Therefore, butyrate exerts anticancer effects by modulating multiple signaling pathways with both HDAC-inhibition and HDAC-independent mechanisms.

While butyrate is well demonstrated to have anti-cancer effects both in vitro and in vivo, the medical application has not been widely adopted due to the low bioavailability and the short half-life observed in the circulation. Nano-delivery may overcome these disadvantages so that the bioavailability of butyrate can be increased. Moreover, nano-delivery can significantly facilitate the combinational use of butyrate with other anti-cancer agents and phytochemicals.
